# Deep learning-enabled medical computer vision

**DOI:** 10.1038/s41746-020-00376-2

**Published:** 2021-01-08

**Authors:** Andre Esteva, Katherine Chou, Serena Yeung, Nikhil Naik, Ali Madani, Ali Mottaghi, Yun Liu, Eric Topol, Jeff Dean, Richard Socher

**Affiliations:** 1 Salesforce AI Research, San Francisco, CA USA; 2 Google Research, Mountain View, CA USA; 3 Stanford University, Stanford, CA USA; 4 Scripps Research Translational Institute, La Jolla, CA USA

**Keywords:** Health care, Medical research, Computational science

## Abstract

A decade of unprecedented progress in artificial intelligence (AI) has demonstrated the potential for many fields—including medicine—to benefit from the insights that AI techniques can extract from data. Here we survey recent progress in the development of modern computer vision techniques—powered by deep learning—for medical applications, focusing on medical imaging, medical video, and clinical deployment. We start by briefly summarizing a decade of progress in convolutional neural networks, including the vision tasks they enable, in the context of healthcare. Next, we discuss several example medical imaging applications that stand to benefit—including cardiology, pathology, dermatology, ophthalmology–and propose new avenues for continued work. We then expand into general medical video, highlighting ways in which clinical workflows can integrate computer vision to enhance care. Finally, we discuss the challenges and hurdles required for real-world clinical deployment of these technologies.

## Introduction

Computer vision (CV) has a rich history spanning decades^1^ of efforts to enable computers to perceive visual stimuli meaningfully. Machine perception spans a range of levels, from low-level tasks such as identifying edges, to high-level tasks such as understanding complete scenes. Advances in the last decade have largely been due to three factors: (1) the maturation of deep learning (DL)—a type of machine learning that enables end-to-end learning of very complex functions from raw data^2^ (2) strides in localized compute power via GPUs^3^, and (3) the open-sourcing of large labeled datasets with which to train these algorithms^4^. The combination of these three elements has enabled individual researchers the resource access needed to advance the field. As the research community grew exponentially, so did progress.

**Fig. 1:**
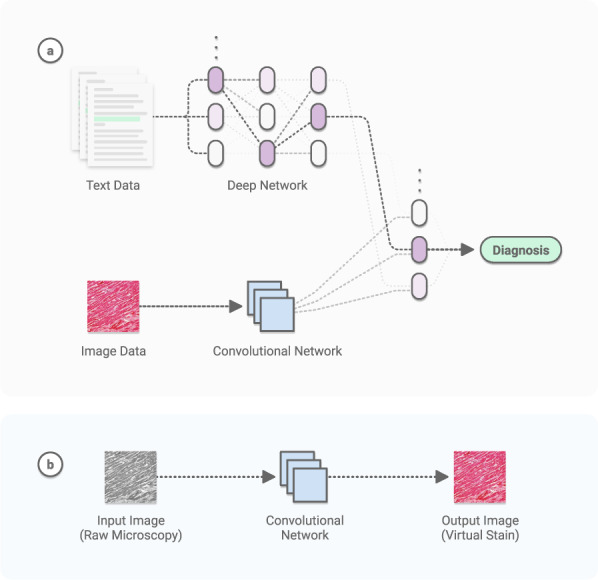
Example medical computer vision tasks. **a** Multimodal discriminative model. Deep learning architectures can be constructed to jointly learn from both image data, typically with convolutional networks, and non-image data, typically with general deep networks. Learned annotations can include disease diagnostics, prognostics, clinical predictions, and combinations thereof. **b** Generative model. Convolutional neural networks can be trained to generate images. Tasks include image-to-image regression (shown), super-resolution image enhancement, novel image generation, and others.

The growth of modern CV has overlapped with the generation of large amounts of digital data in a number of scientific fields. Recent medical advances have been prolific^5,6^, owing largely to DL’s remarkable ability to learn many tasks from most data sources. Using large datasets, CV models can acquire many pattern-recognition abilities—from physician-level diagnostics^7^ to medical scene perception^8^. See Fig. 1.

Here we survey the intersection of CV and medicine, focusing on research in medical imaging, medical video, and real clinical deployment. We discuss key algorithmic capabilities which unlocked these opportunities, and dive into the myriad of accomplishments from recent years. The clinical tasks suitable for CV span many categories, such as screening, diagnosis, detecting conditions, predicting future outcomes, segmenting pathologies from organs to cells, monitoring disease, and clinical research. Throughout, we consider the future growth of this technology and its implications for medicine and healthcare.

## Computer vision

**Fig. 2:**
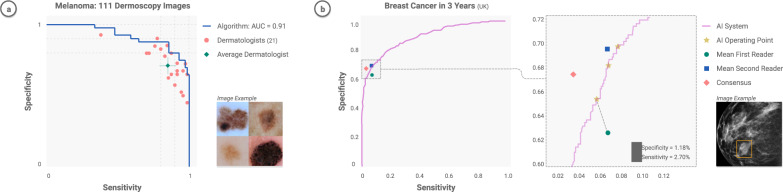
Physician-level diagnostic performance. CNNs—trained to classify disease states—have been extensively tested across diseases, and benchmarked against physicians. Their performance is typically on par with experts when both are tested on the same image classification task. **a** Dermatology^7^ and **b** Radiology^156^. Examples reprinted with permission and adapted for style.

Object classification, localization, and detection, respectively refer to identifying the type of an object in an image, the location of objects present, and both type and location simultaneously. The ImageNet Large-Scale Visual Recognition Challenge^9^ (ILSVRC) was a spearhead to progress in these tasks over the last decade. It created a large community of DL researchers competing and collaborating together to improve techniques on various CV tasks. The first contemporary, GPU-powered DL approach, in 2012^10^, yielded an inflection point in the growth of this community, heralding an era of significant year-over-year improvements^11–14^ through the competition’s final year in 2017. Notably, classification accuracy achieved *human-level* performance during this period. Within medicine, fine-grained versions of these methods^15^ have successfully been applied to the classification and detection of many diseases (Fig. 2). Given sufficient data, the accuracy often matches or surpasses the level of expert physicians^7,16^. Similarly, the *segmentation* of objects has substantially improved^17,18^, particularly in challenging scenarios such as the biomedical segmentation of multiple types of overlapping cells in microscopy. The key DL technique leveraged in these tasks is the convolutional neural network^19^ (CNN)—a type of DL algorithm which hardcodes translational invariance, a key feature of image data. Many other CV tasks have benefited from this progress, including image registration (identifying corresponding points across similar images), image retrieval (finding similar images), and image reconstruction and enhancement. The specific challenges of working with medical data require the utilization of many types of AI models.

These techniques largely rely on supervised learning, which leverages datasets that contain both data points (e.g. images) and data labels (e.g. object classes). Given the sparsity and access difficulties of medical data, transfer learning—in which an algorithm is first trained on a large and unrelated corpus (e.g. ImageNet^4^), then fine-tuned on a dataset of interest (e.g. medical)—has been critical for progress. To reduce the costs associated with collecting and labeling data, techniques to generate synthetic data, such as data augmentation^20^ and generative adversarial networks (GANs)^21^ are being developed. Researchers have even shown that crowd-sourcing image annotations can yield effective medical algorithms^22,23^. Recently, self-supervised learning^24^—in which *implicit* labels are extracted from data points and used to train algorithms (e.g predicting the spatial arrangement of tiles generated from splitting an image into pieces)—have pushed the field towards fully unsupervised learning, which lacks the need for labels. Applying these techniques in medicine will reduce the barrier to development and deployment.

Medical data access is central to this field, and key ethical and legal questions must be addressed. Do patients own their de-identified data? What if methods to re-identify data improve over time? Should the community open-source large quantities of data? To date, academia and industry have largely relied on small, open-source datasets, and data collected through commercial products. Dynamics around data sharing and country-specific availability will impact deployment opportunities. The field of *federated* learning^25^—in which centralized algorithms can be trained on distributed data that never leaves protected enclosures—may enable a workaround in stricter jurisdictions.

These advances have spurred growth in other domains of CV, such as multimodal learning, which combines vision with other modalities such as language (Fig. 1a)^26^, time-series data, and genomic data^5^. These methods can combine with 3D vision^27,28^ to turn depth-cameras into privacy-preserving sensors^29^, making deployment easier for patient settings such as the intensive care unit^8^. The range of tasks is even broader in video. Applications like activity recognition^30^ and live scene understanding^31^ are useful in detecting and responding to important or adverse clinical events^32^.

## Medical imaging

In recent years the number of publications applying computer vision techniques to static medical imagery has grown from hundreds to thousands^33^. A few areas have received substantial attention—radiology, pathology, ophthalmology, and dermatology—owing to the visual pattern-recognition nature of diagnostic tasks in these specialities, and the growing availability of highly structured images.

The unique characteristics of medical imagery pose a number of challenges to DL-based computer vision. For one, images can be massive. Digitizing histopathology slides produces gigapixel images of around 100,000 ×100,000 pixels, whereas typical CNN image inputs are around 200 ×200 pixels. Further, different chemical preparations will render different slides for the same piece of tissue, and different digitization devices or settings may produce different images for the same slide. Radiology modalities such as CT and MRI render equally massive 3D images, forcing standard CNNs to either work with a set of 2D slices, or adjust their internal structure to process in 3D. Similarly, ultrasound renders a time-series of noisy 2D slices of a 3D context–slices which are spatially correlated but not aligned. DL has started to account for the unique challenges of medical data. For instance, multiple-instance-learning (MIL)^34^ enables learning from datasets containing massive images and few labels (e.g. histopathology). 3D convolutions in CNNs are enabling better learning from 3D volumes (e.g MRI and CT)^35^. Spatio-temporal models^36^ and image registration enable working with time-series images (e.g. ultrasound).

Dozens of companies have obtained US FDA and European CE approval for medical imaging AI^37^, and commercial markets have begun to form as sustainable business models are created. For instance, regions of high-throughput healthcare, such as India and Thailand, have welcomed the deployment of technologies such as diabetic retinopathy screening systems^38^. This rapid growth has now reached the point of directly impacting patient outcomes—the US CMS recently approved reimbursement for a radiology stroke triage use-case which reduces the time it takes for patients to receive treatment^39^.

CV in medical modalities with non-standardized data collection requires the integration of CV into existing physical systems. For instance, in otolaryngology, CNNs can be used to help primary care physicians manage patients’ ears, nose, and throat^40^, through mountable devices attached to smartphones^41^. Hematology and serology can benefit from microscope-integrated AIs^42^ that diagnose common conditions^43^ or count blood cells of various types^44^—repetitive tasks that are easy to augment with CNNs. AI in gastroenterology has demonstrated stunning capabilities. Video-based CNNs can be integrated into endoscopic procedures^45^ for scope guidance, lesion detection, and lesion diagnosis. Applications include esophageal cancer screening^46^, detecting gastric cancer^47,48^, detecting stomach infections such as *H. Pylori*^49^, and even finding hookworms^50^. Scientists have taken this field one step further by building entire medical AI devices designed for monitoring, such as at-home smart toilets outfitted with diagnostic CNNs on cameras^51^. Beyond the analysis of disease states, CV can serve the future of human health and welfare through applications such as screening human embryos for implantation^52^.

Computer vision in radiology is so pronounced that it has quickly burgeoned into its own field of research, growing a corpus of work^53–55^ that extends into all modalities, with a focus on X-rays, CT, and MRI. Chest X-ray analysis—a key clinical focus area^33^—has been an exemplar. The field has collected nearly 1 million annotated, open-source images^56–58^—the closest ImageNet^9^ equivalent to date in medical CV. Analysis of brain imagery^59^ (particularly for time-critical use-cases like stroke), and abdominal imagery^60^ have similarly received substantial attention. Disease classification, nodule detection^61^, and region segmentation (e.g. ventricular^62^) models have been developed for most conditions for which data can be collected. This has enabled the field to respond rapidly in times of crisis—for instance, developing and deploying COVID-19 detection models^63^. The field continues to expand with work in image translation (e.g. converting noisy ultrasound images into MRI), image reconstruction and enhancement (e.g. converting low-dosage, low-resolution CT images into high-resolution images^64^), automated report generation, and temporal tracking (e.g. image registration to track tumor growth over time). In the sections below, we explore vision-based applications in other specialties.

## Cardiology

Cardiac imaging is increasingly used in a wide array of clinical diagnoses and workflows. Key clinical applications for deep learning include diagnosis and screening. The most common imaging modality in cardiovascular medicine is the cardiac ultrasound, or echocardiogram. As a cost-effective, radiation-free technique, echocardiography is uniquely suited for DL due to straightforward data acquisition and interpretation—it is routinely used in most acute inpatient facilities, outpatient centers, and emergency rooms^65^. Further, 3D imaging techniques such as CT and MRI are used for the understanding of cardiac anatomy and to better characterize supply-demand mismatch. CT segmentation algorithms have even been FDA—cleared for coronary artery visualization^66^.

There are many example applications. DL can be trained on a large database of echocardiographic studies and surpass the performance of board-certified echocardiographers in view classification^67^. Computational DL pipelines can assess hypertrophic cardiomyopathy, cardiac amyloid, and pulmonary arterial hypertension^68^. EchoNet^69^—a deep learning model that can recognize cardiac structures, estimate function, and predict systemic phenotypes that are not readily identifiable to human interpretation—has recently furthered the field.

To account for challenges around data access,^70^ data-efficient echocardiogram algorithms^70^ have been developed, such as semi-supervised GANs that are effective at downstream tasks (e.g predicting left ventricular hypertrophy). To account for the fact that most studies utilize privately held medical imaging datasets, 10,000 annotated echocardiogram videos were recently open-sourced^36^. Alongside this release, a video-based model, EchoNet-Dynamic^36^, was developed. It can estimate ejection fraction and assess cardiomyopathy, alongside a comprehensive evaluation criterion based on results from an external dataset and human experts.

## Pathology

Pathologists play a key role in cancer detection and treatment. Pathological analysis—based on visual inspection of tissue samples under microscope—is inherently subjective in nature. Differences in visual perception and clinical training can lead to inconsistencies in diagnostic and prognostic opinions^71–73^. Here, DL can support critical medical tasks, including diagnostics, prognostication of outcomes and treatment response, pathology segmentation, disease monitoring, and so forth.

Recent years have seen the adoption of sub-micron-level resolution tissue scanners that capture gigapixel whole-slide images (WSI)^74^. This development, coupled with advances in CV has led to research and commercialization activity in AI-driven digital histopathology^75^. This field has the potential to (i) overcome limitations of human visual perception and cognition by improving the efficiency and accuracy of routine tasks, (ii) develop new signatures of disease and therapy from morphological structures invisible to the human eye, and (iii) combine pathology with radiological, genomic, and proteomic measurements to improve diagnosis and prognosis^76^.

One thread of research has focused on automating the routine, time-consuming task of localization and quantification of morphological features. Examples include the detection and classification of cells, nuclei, and mitoses^77–79^, and the localization and segmentation of histological primitives such as nuclei, glands, ducts, and tumors^80–83^. These methods typically require expensive manual annotation of tissue components by pathologists as training data.

Another research avenue focuses on direct diagnostics^84–86^ and prognostics^87,88^ from WSI or tissue microarrays (TMA) for a variety of cancers—breast, prostate, lung cancer, etc. Studies have even shown that morphological features captured by a hematoxylin and eosin (H&E) stain are predictive of molecular biomarkers utilized in theragnosis^85,89^. While histopathology slides digitize into massive, data-rich gigapixel images, region-level annotations are sparse and expensive. To help overcome this challenge, the field has developed DL algorithms based on multiple-instance learning^90^ that utilize slide-level “weak” annotations and exploit the sheer size of these images for improved performance.

The data abundance of this domain has further enabled tasks such as virtual staining^91^, in which models are trained to predict one type of image (e.g. a stained image) from another (e.g. a raw microscopy image). See Fig. 1b. Moving forward, AI algorithms that learn to perform diagnosis, prognosis, and theragnosis using digital pathology image archives and annotations readily available from electronic health records have the potential to transform the fields of pathology and oncology.

## Dermatology

The key clinical tasks for DL in dermatology include lesion-specific differential diagnostics, finding concerning lesions amongst many benign lesions, and helping track lesion growth over time^92^. A series of works have demonstrated that CNNs can match the performance of board-certified dermatologists at classifying malignant skin lesions from benign ones^7,93,94^. These studies have sequentially tested increasing numbers of dermatologists (25–^7^ 57–^93^, 157–^94^), consistently demonstrating a sensitivity and specificity in classification that matches or even exceeds physician levels. These studies were largely restricted to the binary classification task of discerning benign vs malignant cutaneous lesions, classifying either melanomas from nevi or carcinomas from seborrheic keratoses.

Recently, this line of work has expanded to encompass differential diagnostics across dozens of skin conditions^95^, including non-neoplastic lesions such as rashes and genetic conditions, and incorporating non-visual metadata (e.g. patient demographics) as classifier inputs^96^. These works have been catalyzed by open-access image repositories and AI challenges that encourage teams to compete on predetermined benchmarks^97^.

Incorporating these algorithms into clinical workflows would allow their utility to support other key tasks, including large-scale detection of malignancies on patients with many lesions, and tracking lesions across images in order to capture temporal features, such as growth and color changes. This area remains fairly unexplored, with initial works that jointly train CNNs to detect and track lesions^98^.

## Ophthalmology

Ophthalmology, in recent years, has observed a significant uptick in AI efforts, with dozens of papers demonstrating clinical diagnostic and analytical capabilities that extend beyond current human capability^99–101^. The potential clinical impact is significant^102,103^—the portability of the machinery used to inspect the eye means that pop-up clinics and telemedicine could be used to distribute testing sites to underserved areas. The field depends largely on fundus imaging, and optical coherence tomography (OCT) to diagnose and manage patients.

CNNs can accurately diagnose a number of conditions. Diabetic retinopathy—a condition in which blood vessels in the eyes of diabetic patients “leak” and can lead to blindness—has been extensively studied. CNNs consistently demonstrate physician-level grading from fundus photographs^104–107^, which has led to a recent US FDA-cleared system^108^. Similarly, they can diagnose or predict the progression of center-involved diabetic macular edema^109^, age-related macular degeneration^107,110^, glaucoma^107,111^, manifest visual field loss^112^, childhood blindness^113^, and others.

The eyes contain a number of non-human-interpretable features, indicative of meaningful medical information, that CNNs can pick up on. Remarkably, it was shown that CNNs can classify a number of cardiovascular and diabetic risk factors from fundus photographs^114^, including age, gender, smoking, hemoglobin-A1c, body-mass index, systolic blood pressure, and diastolic blood pressure. CNNs can also pick up signs of anemia^115^ and chronic kidney disease^116^ from fundus photographs. This presents an exciting opportunity for future AI studies predicting nonocular information from eye images. This could lead to a paradigm shift in care in which eye exams screen you for the presence of both ocular and nonocular disease—something currently limited for human physicians.

## Medical video

### Surgical applications

The CV may provide significant utility in procedural fields such as surgery and endoscopy. Key clinical applications for deep learning include enhancing surgeon performance through real-time contextual awareness^117^, skills assessments, and training. Early studies have begun pursuing these objectives, primarily in video-based robotic and laparoscopic surgery—a number of works propose methods for detecting surgical tools and actions^118–124^. Some studies analyze tool movement or other cues to assess surgeon skill^119,121,123,124^, through established ratings such as the Global Operative Assessment of Laparoscopic Skills (GOALS) criteria for laparoscopic surgery^125^. Another line of work uses CV to recognize distinct phases of surgery during operations, towards developing context-aware computer assistance systems^126,127^. CV is also starting to emerge in open surgery settings^128^, of which there is a significant volume. The challenge here lies in the diversity of video capture viewpoints (e.g., head-mounted, side-view, and overhead cameras) and types of surgeries. For all types of surgical video, translating CV analysis to tools and applications that can improve patient outcomes is a natural next direction of research.

### Human activity

**Fig. 3:**
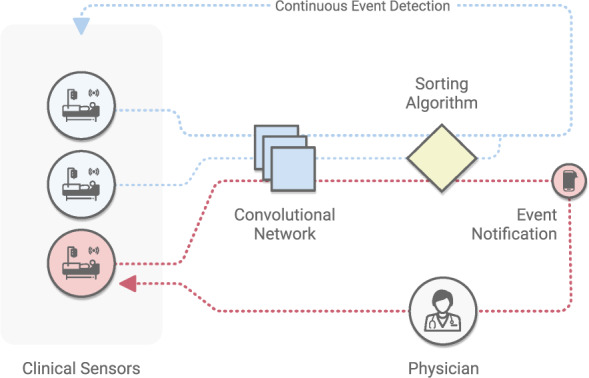
Ambient intelligence. Computer vision coupled with sensors and video streams enables a number of safety applications in clinical and home settings, enabling healthcare providers to scale their ability to monitor patients. Primarily created using models for fine-grained activity recognition, applications may include patient monitoring in ICUs, proper hand hygiene and physical action protocols in hospitals and clinics, anomalous event detection, and others.

CV can recognize human activity in physical spaces, such as hospitals and clinics, for a range of “ambient intelligence” applications. Ambient intelligence refers to a continuous, non-invasive awareness of activity in a physical space that can provide clinicians, nurses, and other healthcare workers with assistance such as patient monitoring, automated documentation, and monitoring for protocol compliance (Fig. 3). In hospitals, for example, early works have demonstrated CV-based ambient intelligence in intensive care units to monitor for safety-critical behaviors such as hand hygiene activity^32^ and patient mobilization^8,129,130^. CV has also been developed for the emergency department, to transcribe procedures performed during the resuscitation of a patient^131^, and for the operating room (OR), to recognize activities for workflow optimization^132^. At the hospital operations level, CV can be a scalable and detailed form of labor and resource measurement that improves resource allocation for optimal care^133^.

Outside of hospitals, ambient intelligence can increase access to healthcare. For instance, it could enable at-risk seniors to live independently at home, by monitoring for safety and abnormalities in daily activities (e.g. detecting falls, which are particularly dangerous for the elderly^134,135^), assisted living, and physiological measurement. Similar work^136–138^ has targeted broader categories of daily activity. Recognizing and computing long-term descriptive analytics of activities such as sleeping, walking, and sitting over time can detect clinically meaningful changes or anomalies^136^. To ensure patient privacy, researchers have developed CV algorithms that work with thermal video data^136^. Another application area of CV is assisted living or rehabilitation, such as continuous sign language recognition to assist people with communication difficulties^139^, and monitoring of physiotherapy exercises for stroke rehabilitation^140^. CV also offers potential as a tool for remote physiological measurements. For instance, systems could use video^141^ to analyze heart and breathing rates^141^. As telemedicine visits increase in frequency, CV could play a role in patient triaging, particularly in times of high demand such as the COVID-19 pandemic^142^. CV-based ambient intelligence technologies offer a wide range of opportunities for increased access to quality care.; However new ethical and legal questions will arise^143^ in the design of these technologies.

## Clinical deployment

As medical AI advances into the clinic^144^, it will simultaneously have the power to do great good for society, and to potentially exacerbate long-standing inequalities and perpetuate errors in medicine. If done properly and ethically, medical AI can become a flywheel for more equitable care—the more it is used, the more data it acquires, the more accurate and general it becomes. The key is in understanding the data that the models are built on and the environment in which they are deployed. Here, we present four key considerations when applying ML technologies in healthcare: assessment of data, planning for model limitations, community participation, and trust building.

Data quality largely determines model quality; identifying inequities in the data and taking them into account will lead towards more equitable healthcare. Procuring the right datasets may depend on running human-in-the-loop programs or broad-reaching data collection techniques. There are a number of methods that aim to remove bias in data. Individual-level bias can be addressed via expert discussion^145^ and labeling adjudication^146^. Population-level bias can be addressed via missing data supplements and distributional shifts. International multi-institutional evaluation is a robust method to determine generalizability of models across diverse populations, medical equipment, resource settings, and practice patterns. In addition, using multi-task learning^147^ to train models to perform a variety of tasks rather than one narrowly defined task, such as multi-cancer detection from histopathology images^148^, makes them more generally useful and often more robust.

**Fig. 4:**
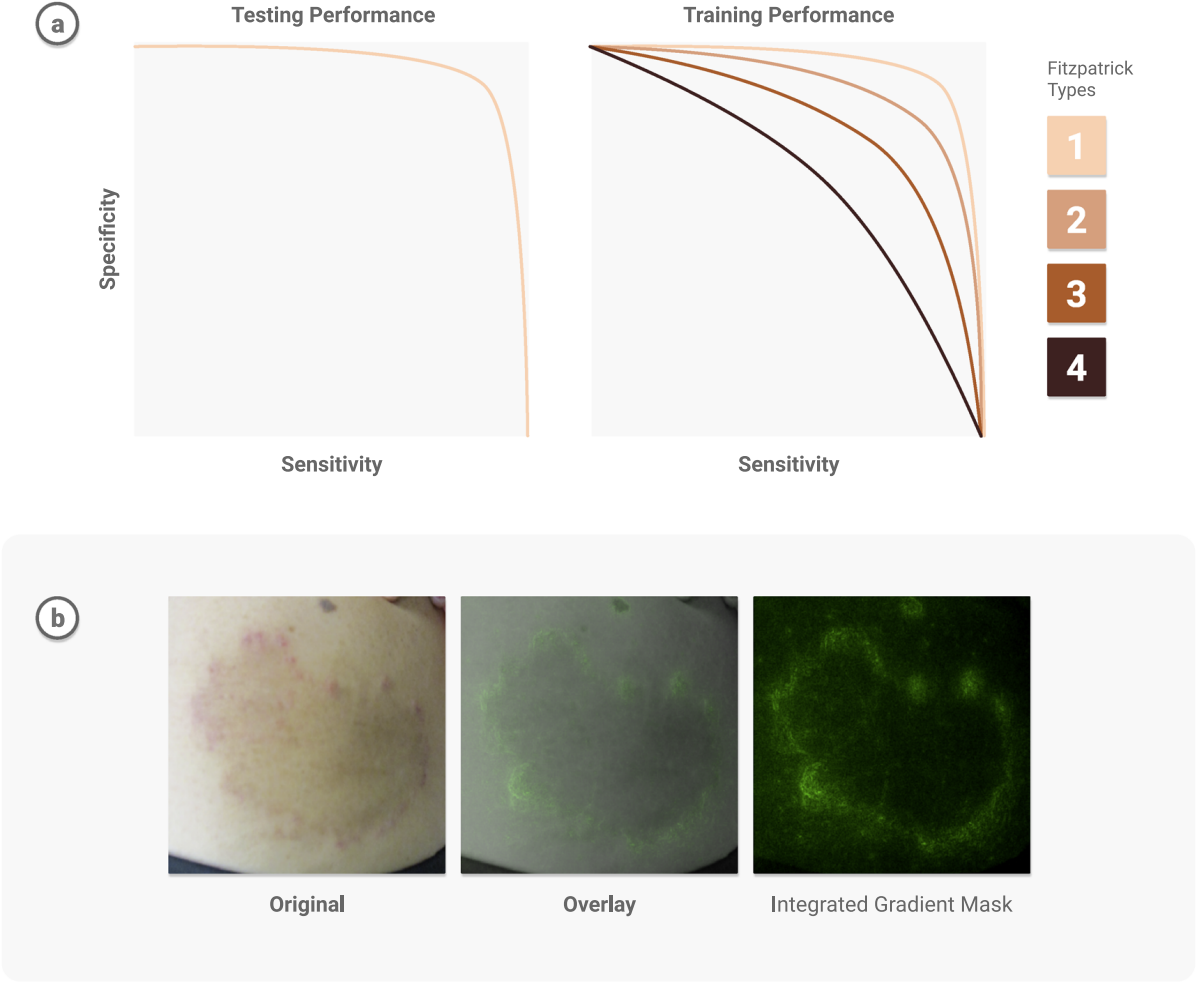
Bias in deployment. **a** Example graphic of biased training data in dermatology. AIs trained primarily on lighter skin tones may not generalize as well when tested on darker skin^157^. Models require diverse training datasets for maximal generalizability (e.g.^95^). **b** Gradient Masks project the model’s attention onto the original input image, allowing practitioners to visually confirm regions that most influence predictions. Panel was reproduced from ref. ^95^ with permission.

Transparent reporting can reveal potential weaknesses and help address model limitations. Guardrails to protect against possible worst-case scenarios—minority, dismissal, or automation bias—must be put in place. It is insufficient to report and be satisfied with strong performance measures on general datasets when delivering care for patients—there should be an understanding of the specific instances in which the model fails. One technique is to assess demographic performance in combination with saliency maps^149^, to visualize what the model pays attention to, and check for potential biases. For instance, when using deep learning to develop a differential diagnosis for skin diseases^95^, researchers examined the model performance based on Fitzpatrick skin types and other demographic information to determine patient types for which there were insufficient examples, and inform future data collection. Further, they used saliency masks to verify the model was informed by skin abnormalities and not skin type. See Fig. 4.

A known limitation of ML is its performance on out-of-distribution data–data samples that are unlike any seen during model training. Progress has been made on out-of-distribution detection^150^ and developing confidence intervals to help detect anomalies. Additionally, methods are developing to understand the uncertainty^151^ around model outputs. This is especially critical when implementing patient-specific predictions that impact safety.

**Fig. 5:**
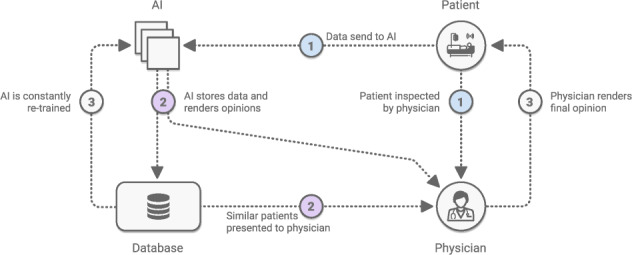
Clinical Deployment. An example workflow showing the positive compounding effect of AI-enhanced workflows, and the resultant trust that can be built. AI predictions provide immediate value to physicians, and improve over time as bigger datasets are collected.

Community participation—from patients, physicians, computer scientists, and other relevant stakeholders—is paramount to successful deployment. This has helped identify structural drivers of racial bias in health diagnostics—particularly in discovering bias in datasets and identifying demographics for which models fail^152^. User-centered evaluations are a valuable tool in ensuring a system’s usability and fit into the real world. What’s the best way to present a model’s output to facilitate clinical decision making? How should a mobile app system be deployed in resource-constrained environments, such as areas with intermittent connectivity? For example, when launching ML-powered diabetic retinopathy models in Thailand and India, researchers noticed that model performance was impacted by socioeconomic factors^38^, and determined that where a model is most useful may *not* be where the model was generated. Ophthalmology models may need to be deployed in endocrinology care, as opposed to eye centers, due to access issues in the specific local environment. Another effective tool to build physician trust in AI results is side-by-side deployment of ML models with existing workflows (e.g manual grading^16^). See Fig. 5. Without question, AI models will require rigorous evaluation through clinical trials, to gauge safety and effectiveness. Excitingly, AI and CV can also help support clinical trials^153,154^ through a number of applications—including patient selection, tumor tracking, adverse event detection, etc—creating an ecosystem in which AI can help design safe AI.

Trust for AI in healthcare is fundamental to its adoption^155^ both by clinical teams and by patients. The foundation of clinical trust will come in large part from rigorous prospective trials that validate AI algorithms in real-world clinical environments. These environments incorporate human and social responses, which can be hard to predict and control, but for which AI technologies must account for. Whereas the randomness and human element of clinical environments are impossible to capture in retrospective studies, prospective trials that best reflect clinical practice will shift the conversation towards measurable benefits in real deployments. Here, AI interpretability will be paramount—predictive models will need the ability to describe *why* specific factors about the patient or environment lead them to their predictions.

In addition to clinical trust, patient trust—particularly around privacy concerns—must be earned. One significant area of need is next-generation regulations that account for advances in privacy-preserving techniques. ML typically does not require traditional identifiers to produce useful results, but there are meaningful signals in data that can be considered sensitive. To unlock insights from these sensitive data types, the evolution of privacy-preserving techniques must continue, and further advances need to be made in fields such as federated learning and federated analytics.

Each technological wave affords us a chance to reshape our future. In this case, artificial intelligence, deep learning, and computer vision represent an opportunity to make healthcare far more accessible, equitable, accurate, and inclusive than it has ever been.

## Data Availability

Data sharing not applicable to this article as no datasets were generated or analyzed during the current study.
